# Analysis of Underwater Melting Process and Leakage Plugging Performance of Phase-Change Materials

**DOI:** 10.3390/ma17112647

**Published:** 2024-05-30

**Authors:** Shenghang Zhang, Lei Tang, Fei Li, Po Li, Yao Sima, Song Zhao

**Affiliations:** 1 The National Key Laboratory of Water Disaster Prevention, Nanjing 210029, China; sxzhang@nhri.cn; 2 Department of Materials and Structures, Nanjing Hydraulic Research Institute, Nanjing 210029, China; lp1129@tju.edu.cn; 3 Jiangsu Provincial Flood Control and Drought Relief Center, Nanjing 210029, China; lifei315@163.com; 4 School of Civil Engineering, Tianjin University, Tianjin 300350, China; 5 China Energy Engineering Group Second Engineering Co., Ltd., Nanchang 330000, China; smy1830529618@163.com (Y.S.); zs15061986298@163.com (S.Z.)

**Keywords:** leakage, self-priming plugging technology, phase-change material, plugging effect, melting process

## Abstract

Leakage is a high-incidence disease of embankment dams, and efficiently addressing this disease guarantees the safe operation of dams. Underwater leakage self-priming plugging technology is a new technology that utilizes the melting and solidifying characteristics of phase-change materials and the negative pressure in the leakage entry area to accurately plug the leakage. However, little is yet known about the underwater melting process of phase-change materials and how their characteristics influence the plugging effect. In this study, three kinds of phase-change materials, namely, paraffin, rosin, and stearic acid, were used to conduct underwater leakage self-priming plugging tests, observe and analyze the underwater melting process, and compare the plugging effects. The results showed that the underwater melting process of phase-change materials exhibited different plugging window periods depending on their melting points, specific heat capacities, and mobilities, which were the main factors affecting their plugging effects. In the final plugging stage, paraffin had the best plugging effect, but the material strength was low; rosin had good plugging compactness, but the fluidity performance was poor, and the material effective utilization was low; stearic acid had a low melting point but dispersed easily. Therefore, a blocking material with a suitable blocking window period can be produced by adjusting the material properties accordingly for an improved blocking effect.

## 1. Introduction

Leakage is a common dike and dam disease which triggers outburst disasters during the flood season [1,2]. The timely adoption of efficient leakage blocking measures is an effective means of preventing dike and dam outburst disasters. In an analysis of 3504 cases of earth dam failures, 35% of the cases were caused by leakage [3]. Dam leakage is the most important factor in a dam failure disaster chain [4]. Currently, leakage sealing measures of embankment dams mainly include geosynthetics covering seepage interception, front bumping slope cultivation, the throwing of soil and rock, grouting, and the pouring of underwater non-dispersed concrete [5,6,7,8,9,10]. These methods have been used in practical engineering projects for many years and have achieved some desirable results. In recent years, frequent occurrences of extreme weather events such as typhoons and heavy rainfall have heightened the probability of dike and dam diseases. The progression from seepage to dam failure can be considered a chain-like process. Timely intervention to address seepage diseases can effectively break the disaster chain and achieve rapid risk mitigation [11,12,13,14]. However, most of the methods are only used to seal the seepage inlet surface, meaning they have little effect on filling the seepage channel inside the dam body; moreover, it is difficult to repair the embankment body seepage at deeper levels from the water level, with the repair efficiency being low [15,16,17]. In terms of the timeliness of rescue operations during the flood season, current efficiency requirements remain insufficiently high.

The relatively new underwater leakage self-priming plugging technology uses phase-change materials to plug dam leakage: the negative pressure suction around the leakage inlet is exploited through the melting of phase-change materials, which are sucked in the liquid state into the leakage channel. Because of the separation of the heat source of the phase-change materials, they are gradually condensed into a solid state in the form of silt, blocking the seepage path and achieving emergency plugging. This technology is characterized by low material consumption and accurate leakage plugging [18,19].

Unlike traditional plugging methods, the phase-change materials used in underwater leakage self-priming plugging technology have higher property requirements. Yet, only a few studies have investigated the plugging characteristics of specialized phase-change materials and the plugging process, which limits the optimization of the ratio of phase-change materials [20]. Therefore, this study investigated three phase-change materials, namely, paraffin [21], rosin [22], and stearic acid [23]; examined their underwater melting process and characteristics; examined their sealing effect on a leakage channel; and analyzed the factors affecting their plugging effect. The findings of this study provide a reference for the subsequent development of high-performance plugging materials that rely on the self-priming blocking technology for underwater leakage.

## 2. Technical Details

### 2.1. Principles of Plugging Methods

**Figure 1:**
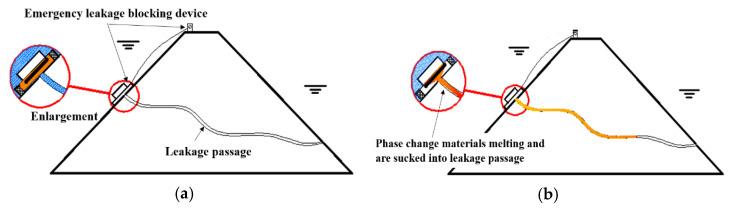
Diagram of leakage sealing process. (**a**) Before sealing the leakage; (**b**) After sealing the leakage.

The process of underwater leakage self-priming plugging technology is illustrated in Figure 1, which is mainly divided into the following two steps: (1) Applying the phase-change materials around the leakage inlet and melting the solid phase-change materials into a liquid. Under the suction force around the leakage inlet, the melted phase-change material is sucked into the leakage channel. (2) After being sucked into the leakage channel, the liquid phase-change material condenses into a solid as its heat is dissipated; the material silts up and blocks the water seepage path, stopping water flow and repairing the leakage channel effectively.

The underwater plugging achieved using this technology is based on solving two problems. The first problem is about heating and melting the phase-change material to a liquid in an infinite water body. The second problem is about the smooth flowing of the liquid plugging material into the leakage channel. Underwater leakage self-priming plugging equipment have been developed to solve these problems and achieve better plugging. Among them, the first problem belongs to thermodynamics, materials science, and other categories [24,25]. The second problem involves the fields of multiphase flow and fluid mechanics [26,27]. At present, there is no device or method that can directly realize the technical purpose of this method, so it is necessary to design and develop relevant devices.

### 2.2. Self-Priming Plugging Equipment

**Figure 2:**
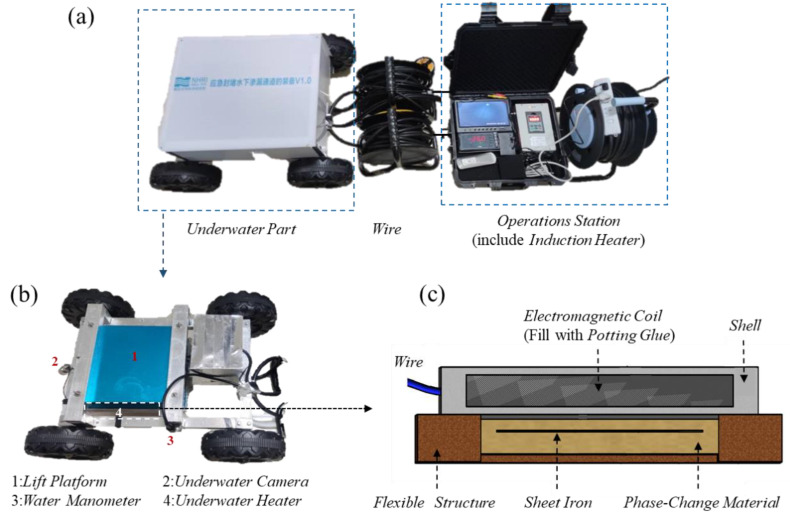
Underwater leakage self-priming plugging equipment. (**a**) Overall structure; (**b**) The underwater part; (**c**) The underwater heating plate.

We developed underwater leakage self-priming plugging equipment using the induction heating principle [28]. As shown in Figure 2, the equipment can be divided into the following two main parts: the water operating platform and the underwater part, and both parts are connected by wires.

The underwater operating table includes an induction heating controller, a display screen, and other components. The underwater part consists of a rack that aids the equipment in traveling and positioning, as well as a device for the underwater heating of the phase-change materials. The frame is equipped with cameras and pressure sensors, which function to place the underwater heating phase-change material device in the leakage inlet perimeter and monitor the water pressure and photography. The underwater heating phase-change material device has a flat-plate structure and consists of an electromagnetic coil, a flexible structure, and a material encapsulation body in three parts [29].

The equipment primarily enables the directional heating of phase-change materials in the underwater environment, which is divided into the following three steps:

(1) The induction heating controller converts ordinary AC power (220 V, 50/60 Hz) into DC power, which is then converted into high-frequency high-voltage power with a frequency of 20–40 KHz. The current is input into the underwater heating plate through the wire.
(2) The underwater heating plate is composed of an electromagnetic coil, a waterproof layer, and a shell. The electromagnetic coil is connected to an electromagnetic heat controller. When the high-frequency high-voltage current with high-speed changes enters the coils, the coil produces an alternating magnetic field with high-speed changes. A waterproof sealant is used to encapsulate the electromagnetic coil, and a dense waterproof layer is formed by setting a hard shell on the outside, enabling the device to function normally underwater.
(3) The material encapsulation body consists of a flux conductor, a flexible rim, and a phase-change blocking material. The flexible edge is a frame structure, which forms a cavity to hold the phase-change material. The flux conductor is embedded in the phase-change material. When the magnetic field generated by the underwater heating plate continuously cuts the flux conductor, large amounts of small eddy currents are generated in the flux conductor, which generates heat and melts the phase-change material in the cavity.

When the equipment is in use, the underwater part is first brought close to the underwater seepage inlet by the control frame, and the lifting platform is used to bring the underwater heated phase-change material unit close to the seepage inlet. Then, the induction heating controller is activated to heat the phase-change material, which melts and flows into the leakage channel. When the material is solidified in the leakage channel, the leakage channel becomes effectively sealed. During this process, the flexible edge protects the melted phase-change material from being dispersed by the water flow.

### 2.3. Phase-Change Materials for Plugging Leaks

Phase-change materials are substances whose state of matter changes and generates latent heat at a constant temperature [30]. In the solid–liquid phase change, for example, the phase change occurs when the material is heated to the melting temperature [31]. During the melting, the phase-change material absorbs and stores a large amount of latent heat; when the material cools down, the stored heat is emitted to the environment within a certain temperature range, and the phase change is reversed from liquid to solid [32]. Currently, phase-change materials are mostly used in the chemical industry, construction, and other fields [33]. In this paper, the phase-change material for plugging leaks (PCMP) refers to phase-change materials used for underwater leakage plugging.

The self-priming plugging technology for underwater seepage channels requires plugging phase-change materials with specific properties:

(1) Density [34]. The phase-change material melting and plugging processes occur underwater, and an excessively low (or high) material density will cause the material to float to the water surface (or sink to the bottom for an excessively high density). This results in material dissipation, reducing the plugging effect. Therefore, the density of phase-change materials used in underwater plugging should be close to that of water.
(2) Melting point and specific heat capacity [35,36,37]. To ensure that the material is rapidly melted into liquid in the underwater environment, the melting point and specific heat capacity of the phase-change material should not be excessively high. Technical requirements stipulate that phase-change plugging materials must be solid at room temperature. Therefore, the melting point of the material should be slightly higher than the room temperature.
(3) Impermeability [38,39]. To achieve a desirable leakage plugging effect, the phase-change material should have good impermeability.
(4) Environmental protection [40,41]. From an ecological point of view, materials that do not pollute waters should be used.

**Figure 3:**
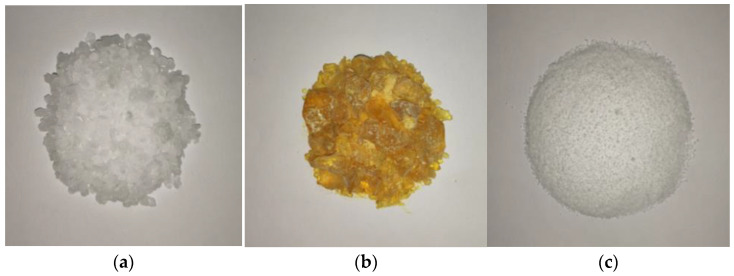
Phase-change blocking materials. (**a**) Paraffin; (**b**) Rosin; (**c**) Stearic acid.

Paraffin, stearic acid, and rosin have densities that are close to the density of water, good impermeability, and suitable melting points [23,42,43]. These materials have demonstrated relatively good plugging effect in self-priming plugging tests of underwater seepage. Therefore, these three materials were used in this study to investigate underwater melting and leakage plugging. The three materials are shown in Figure 3, and the relevant physical parameters are presented in Table 1.

### 2.4. Leakage Plugging Process

The methodology employed in this study focuses on the emergency sealing of centralized leakage channels in dams, contingent upon the identification of the leakage channel entrance. The procedural steps involved are as follows:

(1) Melting the phase-change sealing material within the adaptable structure of the underwater leakage self-priming plugging equipment.
(2) Deploying the underwater leakage self-priming plugging equipment to envelop the phase-change material around the leakage inlet.
(3) Activating the induction heater to induce heat generation within the sheet iron via high-frequency alternating magnetic fields, thereby heating the phase-change material.
(4) Upon liquefaction of the phase-change material, it is drawn into the leakage channel through the suction of the flow field surrounding the leakage inlet, thus achieving effective leakage plugging.

## 3. Test Methods

### 3.1. Test Model

**Figure 4:**
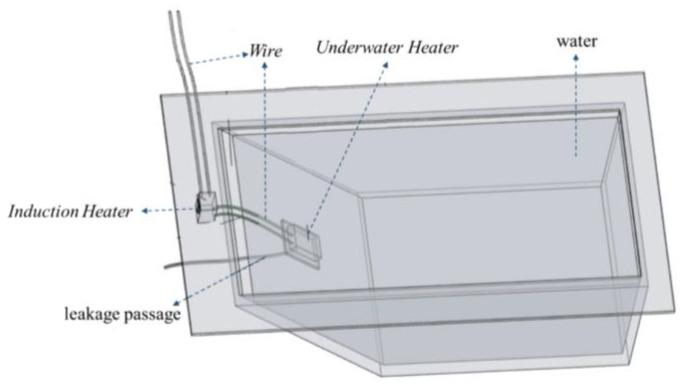
Leakage test model.

As shown in Figure 4, a leakage model was fabricated using plexiglass sheets for indoor testing. The model had an inverted trapezoidal shape, with bottom dimensions of 0.3 cm × 0.4 m, top dimensions of 0.4 m × 0.7 m, and a height of 0.4 m. The angle of the leakage slope was 45°. The leakage slope had a leakage hole with a diameter of 15 mm, and a water pipe was connected to the outside of the leakage hole to simulate the leakage channel. A water inlet was set on the other side of the model, and a water valve was installed on the model to adjust the inlet flow by controlling the water valve. A water-stopping clip was set on the water pipe to adjust the leakage flow by adjusting the stopping clip and replacing the water pipe with different aperture diameters. As the indoor test conditions were more ideal, it was unnecessary to perform location and other operations. Hence, the test only used underwater heating phase-change materials and related devices such as an induction heater and underwater heater. The induction heater was a high-frequency high-voltage electric-input heater. The internal iron was heated through the cutting and changing of the magnetic field to generate heat. The induction heater generates heat from the changing magnetic field, which melts the phase-change material.

### 3.2. Observation Process

The shell of the test model was made of a transparent plexiglass plate, through which the underwater melting of the phase-change material was clearly observed when the underwater heater was in place. As the camera was set on one side of the test model, and with the lens facing the heater placed on the slope, the camera could record the melting process of the phase-change material underwater.

When the plugging was completed, the phase-change material in the leakage channel (the part of the material that plays the plugging role) was collected in situ, and the plugging characteristics of the different materials were recorded by observing their morphology, mass distribution, and other characteristics.

## 4. Test Results

### 4.1. Underwater Melting Characteristics

To accurately describe the underwater melting process of the material, the plugging device was positioned such that the side with the smaller water head was “upper” while that with the larger water head was “lower”. The side close to the leakage surface was the “material surface layer”, and the demarcation line between the solid and liquid state of the phase-change plugging material was the “melting boundary”. During the test, a camera was used to film the melting and diffusion of the phase-change material.

#### 4.1.1. Underwater Melting Process of Paraffin Waxes

**Figure 5:**
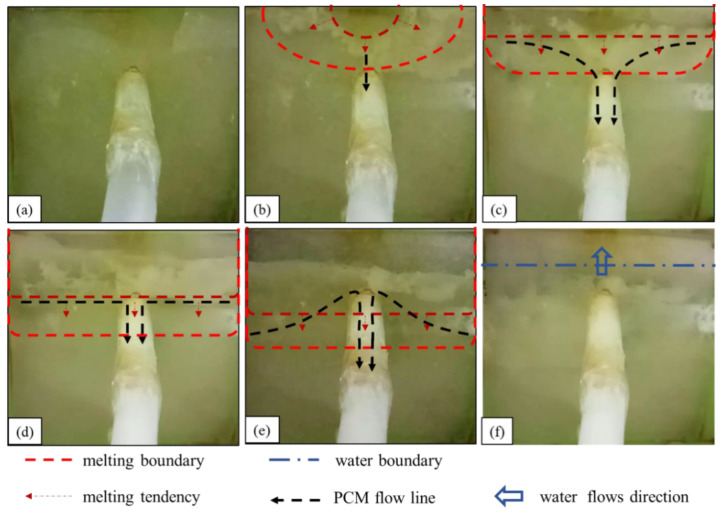
Underwater melting process of paraffin wax: (**a**) before heating; (**b**) liquid paraffin begins to flow into the leakage channel; (**c**) diffused downward melting; (**d**,**e**) “explosive” inflow; (**f**) water flow.

The observed underwater melting and sealing process of the leakage channel of paraffin is summarized as follows:

(1) As shown in Figure 5a, before heating, the underwater heating plate of the paraffin wax is laminated to the seepage surface at the seepage inlet. Because the permeability function of the flexible edge guard does not impede the water flow into the seepage hole, the negative suction around the seepage inlet is maintained, enabling the phase-change material to be drawn into the seepage channel.
(2) As shown in Figure 5b, in the first stage, the upper edge of the paraffin surface layer gradually melts, and a small amount of liquid paraffin is sucked into the leakage channel. However, the amount of paraffin flowing into the leakage channel at this stage is very small and cannot provide a sealing effect. This means the iron sheet inside the device melts the surrounding paraffin, transfers heat outward, and melts the solid paraffin surface layer from the middle of the upper edge of the paraffin.
(3) As shown in Figure 5c, in the second stage, the paraffin wax surface melts gradually from the upper edge downward, “spreading”, more paraffin wax flows into the leakage channel, and the material gradually condenses and adheres to the wall of the leakage channel. Consequently, the leakage flow rate is reduced. The surface of paraffin in the device is set parallel to the heating plate, but the paraffin surface melting is highly asynchronous, i.e., it melts gradually from the top to the bottom, which indicates that the internal paraffin surges after melting underwater.
(4) As shown in Figure 5d,e, in the third stage, the melting line of the paraffin wax gradually moves downward, and when it touches the leakage inlet, a large amount of liquid paraffin wax rushes into the leakage channel, resulting in a long-lasting “explosive” inflow phenomenon. The large amount of paraffin solidifies and blocks the leakage channel, rapidly decreasing the leakage flow. This stage is the main leakage plugging stage of the phase-change material.
(5) As shown Figure 5f, in the fourth stage, the melting boundary no longer moves downward. Even after prolonged reheating, the lower part of the surface paraffin within the material encapsulation still fails to melt, and the inner part of the phase-change material cavity is penetrated by water. Leakage out of the water point stops, or only a slight seepage occurs, marking the completion of the sealing. After water penetrates the phase-change material cavity, the heating object is enveloped by water. The above process is the heat transfer mechanism of the heating object to the phase-change material.

#### 4.1.2. Underwater Melting Process of Rosin

**Figure 6:**
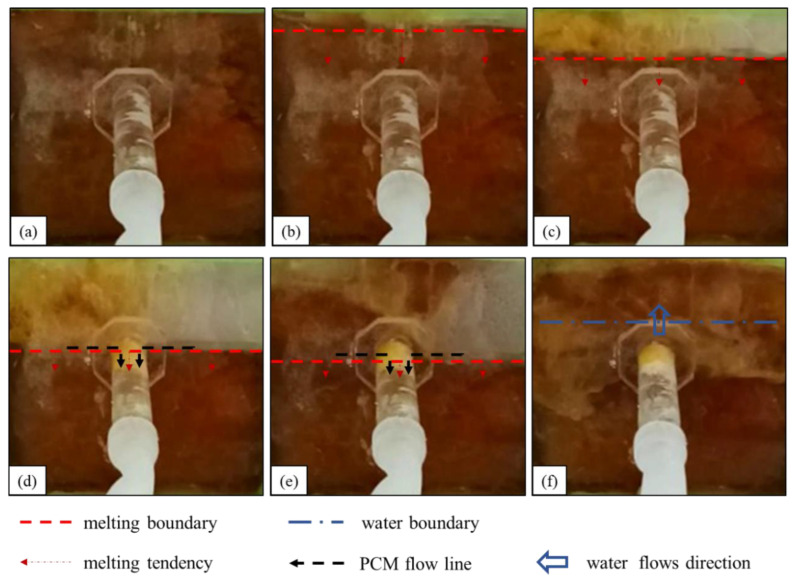
Melting process of rosin under water: (**a**) before heating; (**b**,**c**) diffused downward melting; (**d**,**e**) a small amount of liquid rosin flows into the leakage channel; (**f**) water flow.

The observed process of rosin material melting and sealing the leakage channel underwater is summarized as follows:

(1) The state before heating is shown in Figure 6a. The water flows into the seepage channel, and the negative pressure suction around the seepage inlet is maintained.
(2) The first stage of the rosin water melting process is shown in Figure 6b. The underwater melting process of rosin is similar to that of paraffin, which also melts gradually at the upper edge. However, unlike the paraffin underwater melting, the ring-shaped melting boundary is absent in the rosin case, and rosin does not flow into the leakage channel.
(3) The second stage of the rosin downward melting process is shown in Figure 6c. The difference between the rosin and paraffin melting is that (1) paraffin gradually spreads through downward melting in a smooth process, whereas rosin melting is rapid; (2) in this stage of melting, rosin does not flow smoothly into the seepage channel but rather adheres to the seepage slope.
(4) The third stage of the rosin underwater melting process is shown in Figure 6d,e. When the rosin melting boundary touches the entrance of the seepage channel, a small amount of the liquid rosin is sucked into the seepage channel, and a larger amount of molten rosin accumulates and solidifies around the entrance of the seepage channel. This process is prolonged, during which the flow rate at the leakage outlet is substantially reduced.
  This observation suggests that the higher viscosity and faster solidification of rosin make the material solidify in a shorter period of time after it is removed from the heat source. As such, it only solidifies within a small section of the entrance to the seepage channel and impedes the subsequent flow of molten rosin into the seepage channel.
(5) The fourth stage of the rosin underwater melting process is shown in Figure 6f. A larger amount of molten rosin adheres to and is solidified on the surface around the leakage inlet. Water gradually penetrates the interior of the phase-change material cavity, and the heating of rosin fails.

#### 4.1.3. Underwater Melting Process of Stearic Acid

**Figure 7:**
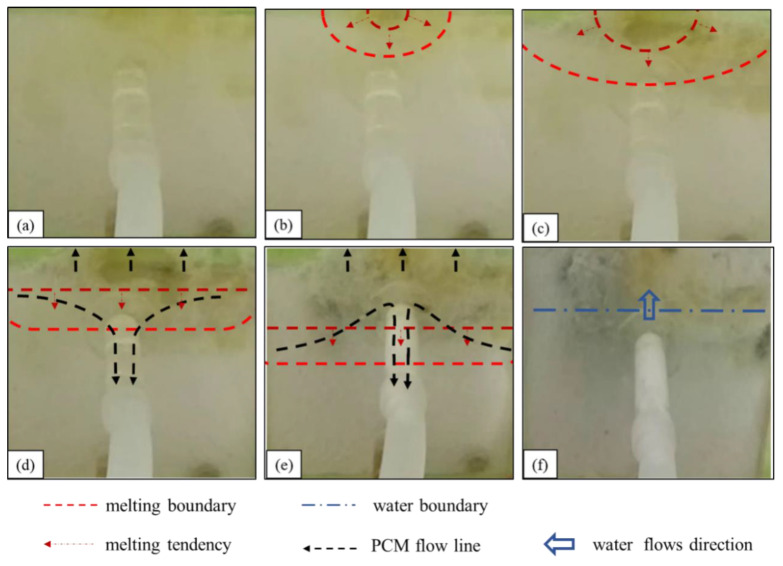
Melting process of stearic acid under water: (**a**) before heating; (**b**,**c**) diffused downward melting. (**d**,**e**) A small amount of liquid stearic acid flows into the leakage channel; (**f**) water flow.

(1) The state of stearic acid before heating is shown in Figure 7a. Water flows into the seepage channel, and the negative pressure suction around the seepage inlet is maintained.
(2) The first stage of the underwater melting of stearic acid is shown in Figure 7b,c. The stearic acid melting starts at the upper edge of the stearic acid, and the melting area gradually expands to both sides and the entire area. This stage of the melting process is similar to that of paraffin but lasts longer.
(3) The second stage of the underwater melting of stearic acid is shown in Figure 7d. The melting boundary extends slowly downward, and some of the molten stearic acid solidifies on the seepage surface with a small amount of stearic acid reaching the surface.
(4) The third stage of the underwater melting of stearic acid is shown in Figure 7e. When the stearic acid melting boundary touches the leakage channel entrance, the stearic acid suddenly rushes into the leakage channel and solidifies rapidly, and the leakage flow rapidly becomes smaller. After the leakage inlet is blocked, the negative pressure suction around the leakage inlet is greatly reduced. The subsequent re-melted stearic acid hardly flows into the leakage channel, and most of it is solidified and bonded to the leakage surface.
(5) The fourth stage of the underwater melting of stearic acid is shown in Figure 7f. Water penetrates the phase-change material cavity, and the heating of the stearic acid fails. After the stearic acid blocks the leakage channel, the leakage is substantially reduced relative to the pre-blocking period, although a slight leakage persists.

Therefore, despite the excellent flow properties of stearic acid, most of the material failed to act as a plug because of its quick solidification on the leakage surface.

### 4.2. Leakage Plugging Effect

#### 4.2.1. Effectiveness of Paraffin Plugging

**Figure 8:**
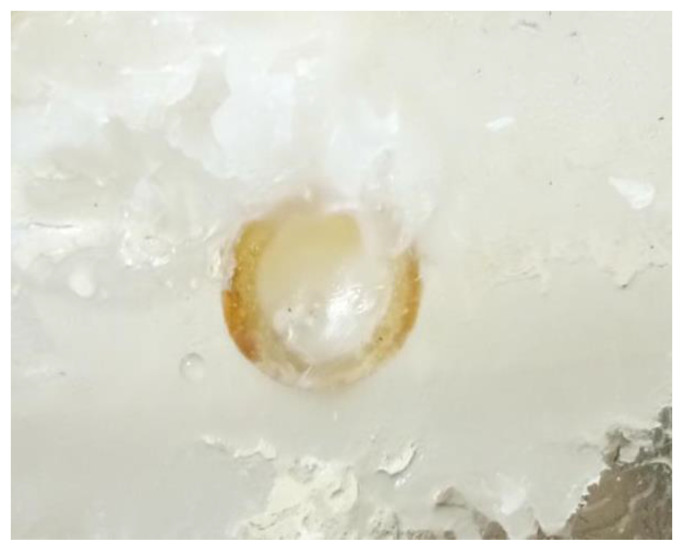
Paraffin plugging effect.

After the liquid paraffin wax flows into the leakage channel, the heat is gradually dissipated, and the material solidifies because it is separated from the heat source. Initially, the solidified paraffin wax is of a small amount, is mostly flocculent or adhered to the pipe wall, and has a small leakage plugging effect (corresponding to the first and second stages of the paraffin wax melting process). When a large amount of liquid paraffin wax flows into the leakage channel, because of the rapid heat loss, the phase-change material condenses rapidly into a solid. Thus, the material plugs the leakage channel and blocks the leakage (corresponding to the third stage of the paraffin wax melting process). After several indoor tests, we found that paraffin wax as the phase-change blocking material had a stable blocking effect. It took only 3 min from the beginning of heating to complete blocking to achieve stable blocking of the underwater leakage channels, with a complete blocking section at the leakage inlet, long blocking length, and continuous inflow into the leakage channel (e.g., Figure 8).

#### 4.2.2. Effectiveness of Rosin Plugging

**Figure 9:**
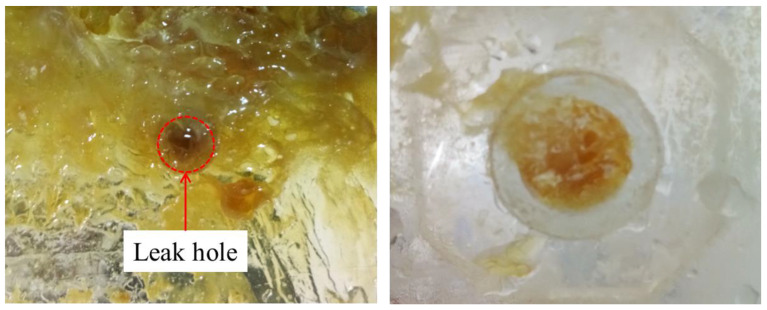
Rosin plugging effect.

When rosin was used as the plugging material, from the start of the heating to complete plugging lasted only 2 min. Rosin was sticky and poorly fluid after melting, and thus a large amount of rosin adhered to the seepage surface (corresponding to the second stage of rosin melting). A small amount of rosin flowed into the seepage channel, and the plugging length was relatively short (corresponding to the third stage of the rosin melting process). This result suggests that rosin is less fluid and has a shorter melting time window. However, the rosin leakage channel seal is relatively dense, has no holes, and is close to the pipe wall; moreover, the material is hard, which can achieve a better seepage prevention effect (e.g., Figure 9).

#### 4.2.3. Effectiveness of Stearic Acid in Plugging Leaks

**Figure 10:**
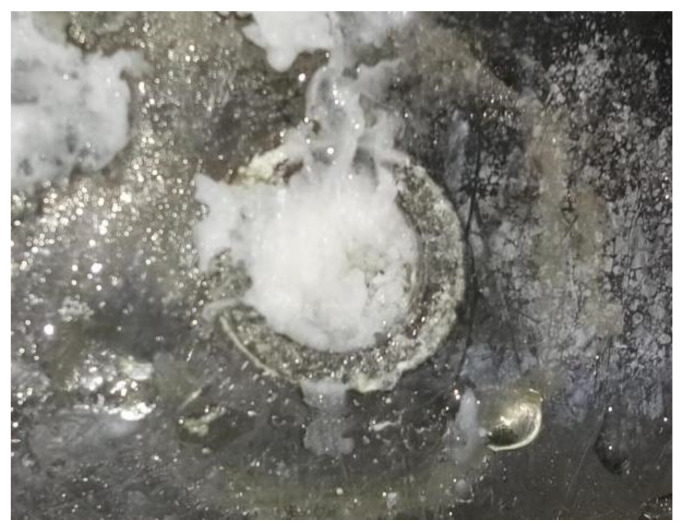
Stearic acid plugging effect.

When stearic acid was used as the plugging material, from the start of the heating to complete plugging lasted for approximately 2.5 min, and a large amount of the material adhered to the entrance of the leakage channel. The material adhered tightly to the pipe wall after solidification and had no holes; however, the end of the plugging length only adhered lightly to the pipe wall, and the material was mostly like a pile of particles, having more holes. The plugging length was slightly longer than that of rosin but shorter than that of paraffin wax. A small amount of stearic acid flowed into the leakage channel, part of the stearic acid adhered to the leakage surface, and the stearic acid floated out during the heating process. Overall, a better plugging effect was realized (e.g., Figure 10).

## 5. Discussion

### 5.1. Characterization of Phase-Change Material Melting

#### 5.1.1. Typical Melting Stage Division

The description in Section 4.1 shows that the underwater melting processes of the three types of phase-change materials share certain similarities and differences. In general, the three processes have the same behavior, as follows:

(1) During the melting process, the surface of the phase-change material always spreads, melting gradually downward from the upper part;
(2) When water penetrates the material filling cavity of the underwater heating equipment, the heat transfer fails, and the phase-change material within the material encapsulation does not melt even if it is reheated for a longer period;
(3) The longitudinal profile of the residual material is “wedge-shaped”: it is thin at the top and gradually thickens downward.

**Figure 11:**
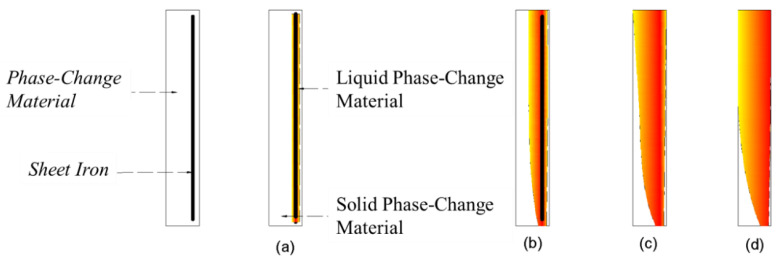
Typical stages of underwater melting of phase-change materials. (**a**) Initial stage; (**b**) Expansion phase; (**c**) Wall-breaking stage; (**d**) Spreading phase.

The observed melting of the surface layer of the material is referred to as the “wall-breaking” phenomenon. When the phase-change plugging material in the material filling cavity “breaks through the wall”, the part of the phase-change material in contact with the iron sheet first melts, and heat is conducted to the surroundings; because the material “breaks through the wall”, it is subject to the buoyancy of the flow field and suction and emerges out of the water surface or flows to the leakage mouth. When the melting line touches the leakage channel, the material flows into the leakage channel, and a large amount of the material in liquid state flows into the leakage channel. When the melting line touches the leakage channel, the material flows rapidly into the leakage channel as a liquid. This process is the key stage that ensures the effective sealing of the leakage channel. According to the behavior observed through the tests, combined with the basic heat transfer law, the melting process can be divided into the following four stages:

(1) Initial stage. When the underwater leakage self-priming plugging equipment is activated, the electromagnetic coil inside the underwater heating plate generates a high-speed changing magnetic field, and the iron sheet inside the phase-change material rapidly heats up because of the cutting of magnetic inductance lines. During this phase, the iron sheet rapidly heats up and melts the surrounding material (Figure 11a).
(2) Expansion phase. At this stage, the molten phase-change material conducts heat to the outer solid phase-change material, and the molten material gradually expands in all directions. Both sides of the material spread to the edges of the pore; the bottom of the liquid phase-change material and water contact, the lower part of the formation of the water-filled area, the lower part of the iron sheet, and the phase-change material heat conduction failure; liquid phase-change material upwelling, the upper part of the phase-change material to the surface layer of the melting, part of the phase change material penetration into the flexible edge of the pore, and solidification (Figure 11b).
(3) Wall-breaking stage. The liquid phase-change plugging material accumulates in the upper part of the material encapsulation body, and the phase-change material on the outer edges begins to melt (“breaking through the wall”). After “breaking the wall”, the material flows to the surface because of the suction and buoyancy or flows into the leakage channel. Part of the molten material remains in the material filling cavity (Figure 11c).
(4) Spreading phase. The surface of the phase-change material spreads downward and melts until the melting boundary touches the leakage channel. A large amount of the material is “sucked” into the leakage channel, and the water medium flows rapidly into the material filling cavity. When water fills the space between the iron sheet and the solid phase-change material, the heat transfer between both fails, resulting in material residue in the device (Figure 11d).

#### 5.1.2. Comparison of Underwater Melting Processes

**Figure 12:**
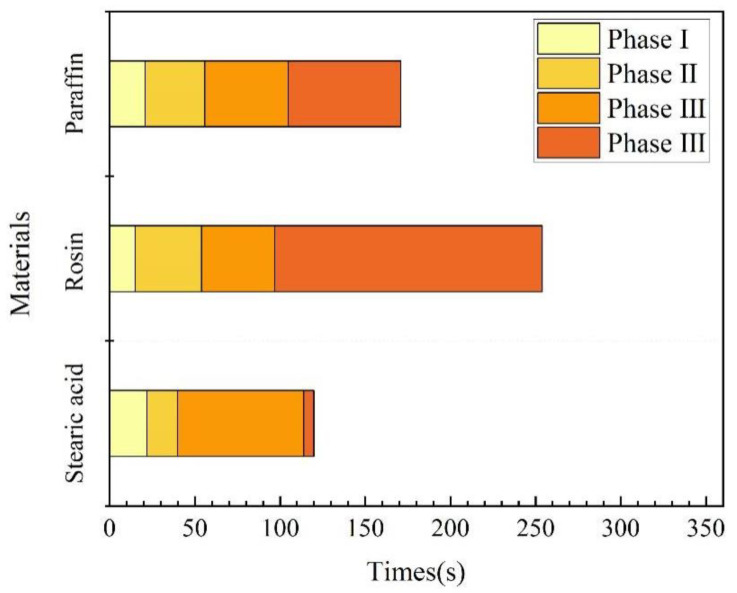
Time for underwater melting of phase-change materials.

The description of the underwater melting of each material in Section 4.1 shows both similarities and differences in the underwater melting processes of the three phase-change materials. The differences are as follows:

(1) Underwater melting time. As mentioned previously, the time taken from the start of heating to the end of heating is referred to as the melting time. A comparison of the melting times of the three materials is illustrated in Figure 12. Rosin had the longest underwater melting time, followed by paraffin wax and then stearic acid. The same test device was used for underwater melting of the three materials, and the material filling volume and heating power were also the same. Therefore, the difference in their underwater melting times mainly stemmed from their respective physical parameters; the main parameters affecting their melting are the specific heat capacity and melting point. In addition, the melting times required for each stage of the three materials were different, which was mainly because the materials became liquid after underwater melting, in addition to their fluidity. For the third stage, which is the key to blocking, stearic acid took the most time to melt, followed by paraffin wax, and then rosin, which is positively correlated with its fluidity performance.

The performance parameters of the three materials are presented in Table 1, from which we can calculate the energy required to reach the melting point or boiling point per unit volume. The formulae are expressed as Equations (1) and (2). Figure 12 illustrates the energy required for each type of material to reach the melting and boiling points. Meanwhile, after heating the material to the melting point, the phase-change material exhibits flow properties; after reaching the boiling point, the material vaporizes. Therefore, only when the material is heated between the melting and boiling points can it flow into the leakage channel, which we call the plugging window, denoted by W in the figure.

(1) $$Q_{m}=\frac{\rho {∆T}_{m}c}{1000}$$

(2) $$Q_{b}=\frac{\rho {∆T}_{b}c}{1000}$$

where $Q_{m}$ is the energy required per unit volume of the phase-change material to reach the melting point, J; $Q_{b}$ is the energy required per unit volume of phase-change material to reach the boiling point, J; ρ is the density of the phase-change material, g/cm^3^; ${∆T}_{m}$ is the temperature difference between the melting point of the phase-change material and the room temperature; ${∆T}_{b}$ is the temperature difference between the boiling point of the phase-change material and the room temperature; c is the specific heat capacity, J·kg^−1^·K^−1^.

**Figure 13:**
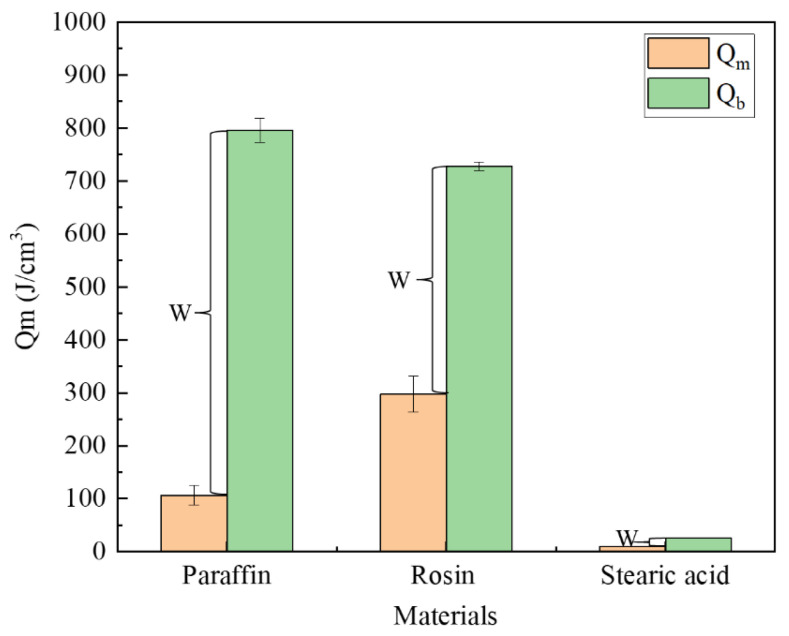
Comparison of energies required.

As shown in Figure 13, rosin required the largest amount of energy to reach the melting point, followed by paraffin, and then stearic acid. The paraffin material has the longest plugging window, followed by rosin and stearic acid. The plugging window represents the energy required for the material to vaporize from the newly attained liquid state, and to a certain extent, it characterizes the length of time during which the material can maintain a liquid form after it is removed from the heat source, that is, the size of the window period left for the material to be sucked into the seepage channel. As found through the tests, the largest amount of paraffin is drawn into the leakage channel, followed by rosin, and then stearic acid. Thus, the rich window period can improve the effective utilization rate of the plugging material.

(2) Cases of the melting boundary being above the leakage inlet. When the melting boundary is above the leakage entrance, a small amount of paraffin is sucked into the leakage channel, rosin is not sucked in, and a small amount of stearic acid floats out of the water. This is mainly because the conversion of the three types of materials from solid to liquid phases changes the density of the material, the liquid state of the material group at the same time by the buoyant force and suction, three types of materials after the phase change, and paraffin and rosin densities are close to that of water, while stearic acid density is small, so a small amount of material appears to surface through the pores of the flexible edge guard.

(3) The melting boundary being below the leakage inlet. When the melting boundary is around the leakage inlet, paraffin appears to flow rapidly into the leakage channel; a small amount of rosin flows into the leakage channel, with most of the material adhering to the slope surface; stearic acid mostly flows into the leakage channel in the early stage, with most of the material adhering to the slope surface in the later stage. This phenomenon occurs mainly because of the different time windows and fluidities of the materials in the liquid state. Rosin and stearic acid have better mobility: when the melting line is around the leakage inlet, both materials are sucked into the leakage channel. However, stearic acid is less dense, and when the leakage channel is blocked and the suction force decreases, it becomes difficult to suck in the material again. Moreover, the shorter condensation window causes the material to solidify faster, with a part of it adhering to the slope surface. Similarly, rosin is less mobile, its solidification point is higher, and the window period is shorter. The rosin material that melts far away from the leakage opening has a relatively short “window period” to flow into the leakage channel; hence, the amount sucked in is less, with a larger amount adhering to the slope surface.

### 5.2. Plugging Characteristics

Relying on the electromagnetic underwater blocking device in the indoor test, paraffin, rosin, and stearic acid phase-change plugging materials effectively blocked the underwater leakage channel. However, the different melting points, specific heat capacities, mobilities, and material densities resulted in different states of leakage channel blocking. The paraffin material had the most stable blocking effect, the longest blocking length, and the best water-stopping effect; the rosin material had the shortest blocking length, the material was brittle, the blocking area was more centralized, and its water-stopping effect was obvious; the stearic acid material had a shorter solidification time, and although it achieved a certain leakage blocking effect, it was difficult to control, leading to incomplete blocking and a low blocking efficiency. Based on the above indoor test results, the advantages and disadvantages of using paraffin, rosin, and stearic acid materials for leakage plugging are summarized in Table 2.

## 6. Conclusions

Underwater leakage self-priming plugging technology was used to investigate the underwater melting process and plugging performance of phase-change materials. Experiments were conducted to study the underwater plugging behavior of paraffin, rosin, and stearic acid as phase-change materials. The characteristics of the underwater melting and the influencing factors were comparatively analyzed, and the plugging effect of the three materials was summarized. The main conclusions are as follows:

(1) According to the characteristics of the underwater melting process of paraffin, rosin, and stearic acid, the process can be summarized into the following four typical stages: initial, expansion, wall-breaking, and spreading stages. The underwater melting process of the phase-change materials depends on the melting point, specific heat capacity, and mobility. These factors vary with the plugging window period, which is the main factor affecting the plugging effect.
(2) Combined with the underwater melting process, the plugging effect of the three phase-change materials was analyzed: paraffin had the best plugging effect, but the material strength is low; rosin has a better plugging compactness, but the flow performance is poor, and the material has a low effective plugging rate; stearic acid is easy to dissipate, but the melting point is low.
(3) In a follow-up study, from the melting point, density, mobility, strength, plugging window period, and other aspects of regulating the deployment of phase-change materials, the formation of better performance of the composite phase-change materials in order to improve the effect of self-absorption plugging of underwater leakage.

## 7. Outlook

The subsequent phase of this methodology will entail exploration across several key domains:

(1) While the research test device has made initial strides in addressing challenges related to underwater phase-change material melting and ensuring negative pressure suction around the leakage inlet, there remains a limitation in the quantity of materials that can be carried at once. Further enhancements are necessary to meet the demand for a continuous material supply.
(2) This study delves into the underwater melting process and plugging efficacy of three types of phase-change materials. However, targeted material design tailored to specific plugging requirements is imperative for further refinement.
(3) While this study has yielded promising outcomes within the laboratory setting, its application and efficacy in practical engineering scenarios necessitate ongoing observation and evaluation.

## Figures and Tables

**Table 1:** Material-related parameters.

Phase-Change Material	Density/g·cm^−3^	Fusion Point/°C	Boiling Point/°C	Specific Heat/J·kg^−1^·K^−1^
Paraffin	0.88–0.915	47–64	412–420	2130
Rosin	1.060–1.085	110–135	300	2260.8
Stearic acid	0.9408	67–69	183–184	149.7

**Table 2:** Characteristics of phase-change materials for leakage plugging.

Material	Paraffin	Rosin	Stearic
Advantage	Long blocking length, suitable melting point, stable, easy to control, good fluidity, and suitable viscosity.	Harder, better plugging compactness.	Small volume shrinkage after liquid–solid transformation, suitable melting point, and excellent fluidity.
Disadvantage	Lower strength, volume contraction of the material after solidification, and uneven distribution of material along the blockage length.	Poor mobility, easy to change state through heating; brittle, easy to age.	Small specific heat capacity, fast solidification, short plugging length, and difficult to control.

## Data Availability

The raw data supporting the conclusions of this article will be made available by the authors on request.
